# Thrombospondin 2 is a Functional Predictive and Prognostic Biomarker for Triple-Negative Breast Cancer Patients With Neoadjuvant Chemotherapy

**DOI:** 10.3389/pore.2022.1610559

**Published:** 2022-08-30

**Authors:** Yuxiang Lin, E. Lin, Yan Li, Xiaobin Chen, Minyan Chen, Jun Huang, Wenhui Guo, Lili Chen, Long Wu, Xiang Zhang, Wenzhe Zhang, Xuan Jin, Jie Zhang, Fangmeng Fu, Chuan Wang

**Affiliations:** ^1^ Department of Breast Surgery, Fujian Medical University Union Hospital, Fuzhou, China; ^2^ Department of General Surgery, Fujian Medical University Union Hospital, Fuzhou, China; ^3^ Breast Cancer Institute, Fujian Medical University, Fuzhou, China; ^4^ School of Cancer and Pharmaceutical Sciences, Translational Oncology and Urology Research (TOUR), King’s College London, London, United Kingdom; ^5^ Department of Laboratory Medicine, Fujian Medical University Union Hospital, Fuzhou, China; ^6^ Department of Pathology, Fujian Medical University Union Hospital, Fuzhou, China; ^7^ Department of Pathology, Fujian Medical University Union Hospital Pingtan Branch, Fuzhou, China

**Keywords:** biomarker, pathological response, neoadjuvant chemotherapy, triple-negative breast cancer, THBS2

## Abstract

**Background:** Triple-negative breast cancer (TNBC) is characterized by a more aggressive biological behavior and unfavorable outcome. Circulating and histological expression of THBS2 has been demonstrated to be a novel diagnostic and prognostic biomarker in patients with various types of tumors. However, few studies have evaluated the predictive and prognostic value of THBS2 in TNBC specifically.

**Methods:** In total, 185 triple-negative breast cancer patients (TNBC) with preoperative neoadjuvant chemotherapy were enrolled in this study. Serum THBS2 (sTHBS2) level was measured both prior to the start of NAC and at surgery by enzyme-linked immunosorbent assay (ELISA). Histological THBS2 (hTHBS2) expression in patients with residual tumors was evaluated by immunohistochemistry (IHC) staining method. Correlations between variables and treatment response were studied. Kaplan-Meier plots and Cox proportional hazard regression model were applied for survival analysis. Functional activities of THBS2 in TNBC cells were determined by CCK-8 assay, colony formation, wound healing, and transwell assay.

**Results:** Of the 185 patients, 48 (25.9%) achieved pathological complete response (pCR) after completion of NAC. Elevated pCR rates were observed in patients with a lower level of sTHBS2 at surgery and higher level of sTHBS2 change (OR = 0.88, 95%CI: 0.79–0.98, *p* = 0.020 and OR = 1.12, 95%CI: 1.02–1.23, *p* = 0.015, respectively). In survival analysis, hTHBS2 expression in residual tumor was of independent prognostic value for both disease-free survival (HR = 2.21, 95%CI = 1.24–3.94, *p* = 0.007) and overall survival (HR = 2.07, 95%CI = 1.09–3.92, *p* = 0.026). For functional studies, THBS2 was indicated to inhibit proliferation, migration, and invasion abilities of TNBC cells *in vitro*.

**Conclusion:** Our findings confirmed the value of serum THBS2 level to predict pCR for TNBC patients and the prognostic performance of histological THBS2 expression in non-pCR responders after NAC. THBS2 might serve as a promising functional biomarker for patients with triple-negative breast cancer.

## Introduction

Triple-negative breast cancer (TNBC) represents one specific type of breast cancer that lacks the expression of estrogen receptor (ER), progesterone receptor (PR), and human epidermal growth factor receptor-2 (HER2). Although TNBC only accounts for about 15–20% of all breast cancer patients [1], it has a more aggressive biological behavior and unfavorable outcome [2]. The use of surgery, chemotherapy, and radiotherapy is the primary established treatment strategy for triple-negative breast cancer [3, 4].

Neoadjuvant chemotherapy (NAC) refers to the administration of chemotherapy prior to definitive breast surgery. The goals of NAC include rendering inoperable tumors resectable, allowing surgical downstaging for breast conservation, and avoiding complete axillary dissections. In addition to its impact on surgery, the neoadjuvant setting has also become a good platform for individual drug response on TNBC patients and provided new insight into tumor biology. After completing NAC treatment, patients with TNBC could have a higher pathological complete response (pCR) rate than those with other subtypes of breast cancer. Patients who achieved pCR could have a lower risk of relapse or death compared to those who have residual disease after NAC [3, 5,6]. Nevertheless, some TNBC patients might still develop a rapid recurrence that could lead to poor prognosis after systemic therapy, especially for those non-pCR responders [7]. Thus, identification of new clinically applicable biomarkers with predictive and prognostic value for NAC response might be beneficial in the treatment of TNBC.

Thrombospondin 2 (THBS2, also known as TSP-2) is an extracellular matrix glycoprotein of the thrombospondin family which contains five members from THBS1 to THBS5 [8]. It was reported to be involved in multiple biological functions, such as extracellular matrix assembly, angiogenesis, and chondrogenic differentiation [9–11]. Evidence has also suggested that THBS2 could play vital roles in cancer progression and metastasis [12–14]. Circulating and histological expression of THBS2 has been demonstrated to be a novel diagnostic and prognostic biomarker for patients with pancreatic cancer [15–17], distal cholangiocarcinoma [15, 18], lung cancer [19, 20], and colorectal cancer [21, 22]. However, few studies have evaluated its predictive and prognostic value in a neoadjuvant setting or in triple-negative breast cancer specifically. Our previous proteomic study has indicated that THBS2 was highly expressed in TNBC tumor tissues [23]. Therefore, this study was conducted to analyze the clinical utility of THBS2 expression in TNBC patients with neoadjuvant chemotherapy and establish a new strategy for identifying subgroup patients of different risk. Furthermore, a series of *in vitro* experiments were also performed to confirm the functional role of THBS2 in TNBC.

## Material and Methods

### Study Patients

In total, 185 breast cancer patients with preoperative neoadjuvant chemotherapy (NAC) in Fujian Medical University Union Hospital between March 2012 to December 2019 were retrospectively selected in this study. All patients were histologically confirmed with TNBC and received at least six cycles of NAC with anthracycline and taxanes-based regimens. Surgical resection was performed followed by the completion of neoadjuvant chemotherapy. Patients with residual invasive tumor after NAC received oral capecitabine for six or eight cycles as recommended by the treating physician. Radiation therapy was performed at the discretion of the radiologist. Our study was approved by the Research Ethics Committee of Fujian Medical University Union Hospital (2022KY122) and written informed consent was obtained from each participant before inclusion in this study.

### Pathological and Survival Evaluation

In this study, the cut-off value for ER and PR was less than 1% of positive tumor cells with nuclear staining. HER2 was considered as positive when immunohistochemistry (IHC) expression was 3 + or fluorescence *in situ* hybridization (FISH) was positive. Only tumors with negative expression of ER, PR, and HER2 were confirmed with TNBC. Pathological complete response (pCR) was defined as no residual invasive tumor in breast or lymph nodes. Patients only with ductal carcinoma *in situ* (DCIS) were also considered as pCR responders. Pathological evaluation was conducted to measure the continuous RCB index (wherein pathologic complete response has RCB 0; residual disease is categorized as RCB-I, RCB-II, and RCB-III) by two independent experienced pathologists [24]. Disease-free survival (DFS) was defined as the time of diagnosis to the date of disease relapse (with histopathology confirmation or radiological evidence of tumor recurrence). Overall survival (OS) was defined as the time of diagnosis until death from any cause. The last follow-up date was 1 May 2021.

### Enzyme-Linked Immunosorbent Assay and Immunohistochemistry

Blood samples were obtained prior to the start of NAC (at baseline) and at surgery. All serum samples were centrifuged and stored at −80°C until use with no more than two freeze-thaw cycles allowed. Serum THBS2 (sTHBS2) level was measured by a commercially available enzyme-linked immunosorbent assay (ELISA) kit (Cat #DTSP20; R&D). Measurements were performed by strictly following the manufacturer’s instructions and repeated three times, with the final result marked as average level. All non-pCR patients were classified into different TNBC subtypes (LAR, IM, BLIS, MES, and US) with the surgical specimens based on the immunohistochemistry (IHC) staining procedures described previously [25]. Histological THBS2 (hTHBS2) expression for the surgical specimens in non-pCR patients were also evaluated by the IHC staining method. Slides were incubated with THBS2 (ab112543, abcam, 1:500) and a negative control was prepared by the substitution of primary antibody with phosphate-buffered saline (PBS, 5% BSA). The IHC staining scores were independently assessed by two pathologists according to the proportion of stained tumor cells and intensity of cellular staining. The proportion of stained tumor cells was scored from 1 to 4: 1, 0%–25%; 2, 26%–50%; 3, 51%–75% and 4, 75%–100%. The intensity of cellular staining was scored from 0 to 3: 0, no staining; 1, weak staining; 2, moderate staining; and 3, strong staining. The percentage of positive tumor cells and the staining intensity were multiplied to produce a weighted score for each patient. A score of 8–12 was defined as high expression level and a score of 0–7 was defined as low expression.

### Cell Culture and Transfection

Human TNBC MDA-MB-231 and BT-549 cells were obtained from the Cell Bank of Type Culture Collection of The Chinese Academy of Sciences. TNBC cells were cultured in DMEM (HyClone; Cytiva) supplemented with 10% FBS (Gibco) and 1% penicillin and streptomycin solution. Cell culture was maintained in a 37°C incubator with 5% CO_2_. Short hairpin RNA (shRNA) targeting THBS2 and its negative control were subcloned into GV493 lentiviral vector (GeneChem) and named sh-THBS2 and sh-Ctrl. Cells were transfected with lentivirus vectors for 48 h and further selected with 2 μg/ml puromycin. The efficiency of THBS2 knockdown was validated by quantitative real-time PCR (qRT-PCR) and western blot. The target sequences of shRNA were as follows:

shTHBS2-1: 5′-CTG​CGA​CCT​CAT​AGA​CAG​CTT-3′

shTHBS2-2: 5′-CCG​CTT​CGT​GCG​CTT​TGA​CTA-3′

shTHBS2-3: 5′-TTG​CTT​CAG​AAC​GTC​CAC​CTA-3′

shNC: 5′-TTC​TCC​GAA​CGT​GTC​ACG​T-3′

#### Western Blotting

Total protein was extracted by RIPA lysis buffer (Beyotime Bio, Inc.). The protein concentrations were determined with BCA Protein Assay Kits (Beyotime Bio, Inc.). A total of 10 µg of protein was loaded and respectively added to gel for electrophoresis and then transferred to a polyvinylidene difluoride (PVDF) membrane. After blocking with 5% non-fat milk at room temperature for 1 h, primary antibodies were bound overnight at 4°C. The next day, the PVDF membrane was incubated with corresponding secondary antibodies at room temperature for 2 h. Chemiluminescence signals were visualized by the enhanced chemiluminescence (ECL) method with ECL kit (Thermo Fisher Scientific, Inc.) and detected by the ChemiDoc Touch System (Bio-Rad Laboratories Inc.). The primary and secondary antibodies used in this study were listed as follows: anti-THBS2 (ab112543, abcam, 1:100), anti-β-actin (sc-69879, Santa Cruz, 1:5000), Rabbit IgG (#7074, CST, 1:10000), and Mouse IgG (#7076, CST, 1:10000).

### Quantitative Real-Time PCR

Total RNA in MDA-MB-231 and BT-549 cells was extracted with TRIzol reagent following the manufacturer’s instructions (Invitrogen). Complementary DNA was synthesized using PrimeScript RT Master Mix (Takara Bio, Inc.) and qRT-PCR was subsequently performed on a model 7500 Real-Time PCR System (Applied Biosystems) with SYBR Green kit (Takara Bio, Inc.). β-actin gene was detected for normalization of data. The respective forward and reverse primers were listed in Supplementary Table S1. Fold changes of gene expression were calculated by the 2−ΔΔct method; three independent replicates of all biological samples were assessed.

### CCK-8 and Colony Formation Assay

Cell proliferation was detected by cell count kit-8 (CCK8) assay and colony formation assay. For the CCK8 assay, 2000 cells were seeded per well in quadruplicate for 96 well plates. 10ul CCK-8 reagent (DOJINDO) was added to each well and the plates were maintained in a 37°C incubator for 2 h. The absorbance at 450 nm was measured by a microplate reader at indicated time points. For the colony formation assay, cells were plated into 6-well plates at a density of 500 cells per dish and cultured for 2 weeks. Colonies were fixed in methanol for 30 min at room temperature and stained with 1000 ul of crystal violet solution (Sangon Bio, Inc.). A colony was defined as >50 cells and the colony formation rate was assessed by colony number/seeding number.

### Wound Healing Assay

MDA-MB-231 and BT-549 cells were spread at the bottom of 96-well plates and cultured overnight. A line wound was made by scraping 10 ul tips across the confluent cell layer. The floating cells were washed three times with PBS and the serum-free medium was used to maintain the cells. The images were captured at 0 and 24 h with an inverted light microscope.

### Transwell Assay

Transwell assay was performed to assess the invasion. Matrigel (Corning, Inc.) was mixed with serum-free medium and added onto the upper surface of the chambers (Corning, Inc.; 8.0-µm filter), while the lower chambers were filled with medium with 30% FBS. After incubation at 37°C for 24 h, the tumor cells attached on the upper surface were removed with cotton swabs. The cells on the lower surface of the membrane were stained with Giemsa (Sigma-Aldrich) after fixation with methanol. The images of invasive cells were collected with a light microscope (magnification, ×100).

### Statistical Analysis

Correlations among sTHBS2 expression, clinicopathological characteristics, and NAC treatment response were compared by student’s *t*-test (for continuous variables) and chi-squared (χ2) test (for categorical variables). Survival analyses were conducted to explore the relationship between hTHBS2 expression, clinicopathological factors, and survival of non-pCR responders. Logistic regression analysis was also applied to identify independent predictors of treatment response. Survival curves were calculated by the Kaplan-Meier method and analyzed by log-rank test. Cox proportional hazard regression model was used for univariate and multivariate survival analysis. Each experiment was repeated 3 times and presented as mean ± standard deviation (SD). Student’s *t*-test and one-way analysis of variance were conducted for comparisons among the groups. Statistical analyses were performed with Statistical Package for the Social Sciences (SPSS, version 24.0) for Windows (Chicago, United States), with a two-sided *p* value of less than 0.05 considered statistically significant.

## Results

### Patient Characteristics

Of the 185 patients, 48 (25.9%) achieved pathological complete response (pCR). The detailed clinicopathological characteristics are shown in Table 1. The included patients had a median age of 49 years (range, 24–72 years). All patients were diagnosed with stage II and stage III disease, among whom 63 (34.1%) were T3/T4 and 151 (81.6%) had lymph node involvement. Most patients (89.7%) were pathologically diagnosed with invasive ductal carcinoma and 104 (56.2%) had a higher histology grade (Grade III). The Ki67 expression of the biopsy specimen was also evaluated, with 120 patients (64.9%) having Ki67 more than 50%.

**TABLE 1:** Baseline characteristics and pathological complete response (pCR) rate in patients with neoadjuvant chemotherapy (NAC).

Characteristics	Number of patients	Number of pCR		*P* ^a^	*P* ^b^
	No.	No.	%		
Age (years)				0.505	NS
≤50	112	31	27.7		
>50	73	17	23.3		
Clinical T stage at baseline				<0.001	0.035
T0/T1	8	6	75.0		
T2	114	35	30.7		
T3	55	6	10.9		
T4	8	1	12.5		
Clinical N stage at baseline				0.005	NS
N0	34	15	44.1		
N1	102	24	23.5		
N2	11	5	45.5		
N3	38	4	10.5		
Histology				0.106	NS
Invasive ductal carcinoma	166	46	27.7		
Others	19	2	10.5		
Grade				0.175	NS
I + II	81	17	21.0		
III	104	31	29.8		
Ki67 expression at baseline				<0.001	0.002
≤50%	65	2	3.1		
>50%	120	46	38.3		
sTHBS2 at baseline (ng/ml)				0.330	NS^c^
≤24.34	64	16	25.0		
>24.34	64	21	32.8		
sTHBS2 at surgery (ng/ml)				<0.001	0.020^c^
≤17.45	64	27	42.2		
>17.45	64	10	15.6		
sTHBS2 change (ng/ml)				<0.001	0.015^c^
≤5.19	64	10	15.6		
>5.19	64	27	42.2		

a The *p* value was compared among all groups by the Chi-square test.

b The *p* value was compared among all groups by multivariate logistic regression analysis.

c sTHBS2 was calculated in the multivariate analysis as linearly variable.

### sTHBS2 and NAC Treatment Response

**FIGURE 1:**
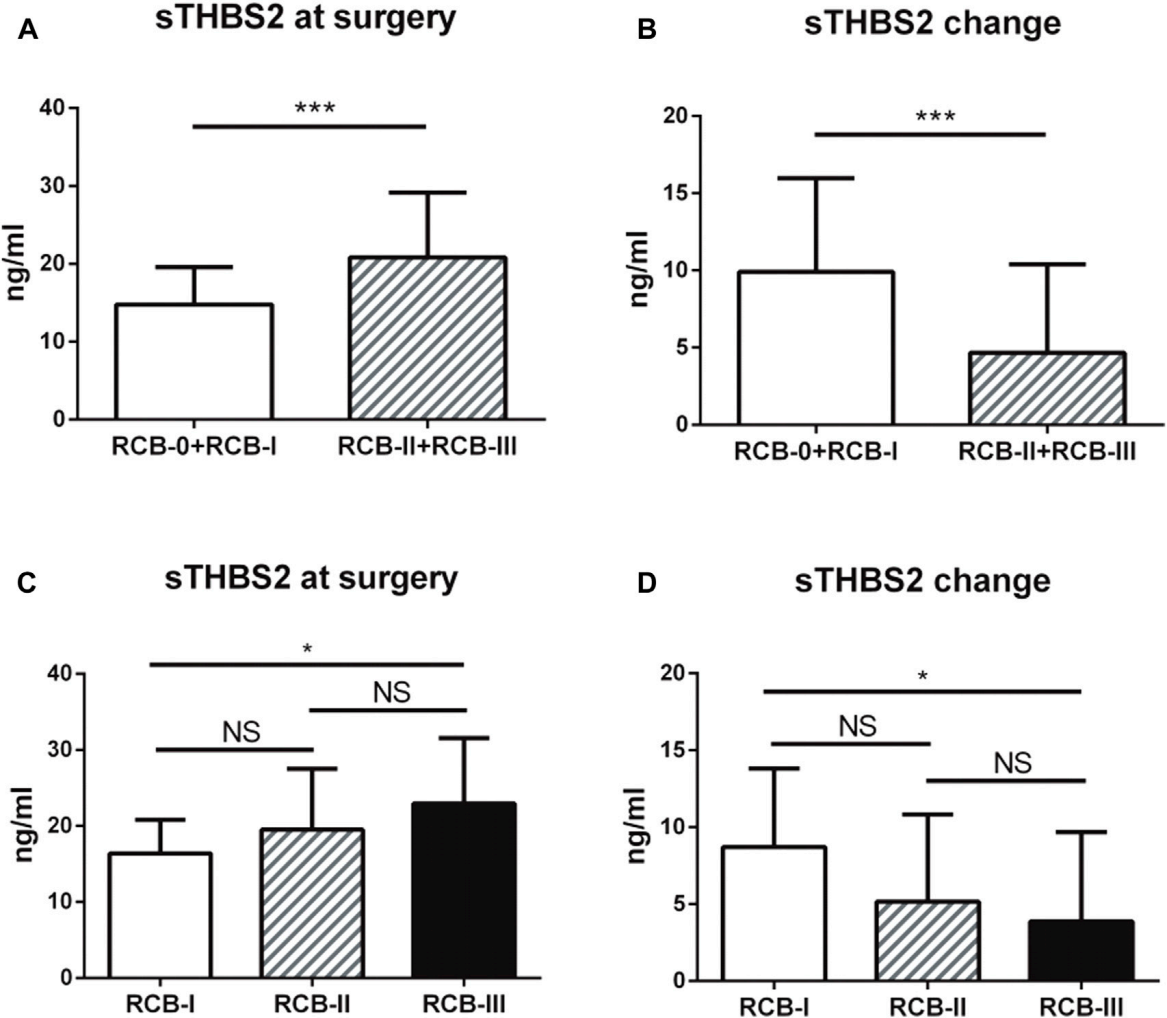
Associations between serum THBS2 (sTHBS2) level and neoadjuvant chemotherapy response. **(A**,**C)** Lower level of serum THBS2 at surgery was correlated with a better treatment response; **(B**,**D)** Higher change level of serum THBS2 during neoadjuvant chemotherapy was correlated with a better treatment response. *, *p* < 0.05; ***, *p* < 0.001.

Serum THBS2 (sTHBS2) was measured by ELISA both prior to the start of NAC (at baseline) and at surgery. Of the patients, 128 (37 pCR and 89 non-pCR) were eligible for serum samples collected at two time points. The median level of sTHBS2 at baseline and surgery was 24.34 ng/ml (range: 8.65 to 47.34 ng/ml) and 17.45 ng/ml (range: 7.49 to 48.13 ng/ml), respectively. The change of sTHBS2 level was also calculated, with the median reduction at 5.19 ng/ml (range: −13.68 to 25.47 ng/ml). The cut-off values of sTHBS2 at baseline, surgery, or change were all categorized by its median level. In chi-squared test, it was demonstrated that lower clinical T stage, lower clinical N stage, higher expression of Ki67, sTHBS2 at surgery, and sTHBS2 change were correlated with higher possibility of achieving pCR. In multivariate logistic regression analysis, sTHBS2 at surgery and the change in sTHBS2 were indicated as independent predictors of pCR as continuous variables (OR = 0.88, 95%CI: 0.79–0.98, *p* = 0.020 and OR = 1.12, 95%CI: 1.02–1.23, *p* = 0.015, respectively). Clinical T sage and Ki67 expression at baseline were also identified to be independent predictors for achieving pCR (*p* = 0.035 and *p* = 0.002, respectively). The correlations among sTHBS2 at surgery, sTHBS2 change, and tumor response (according to the RCB system) are shown in Figure 1. Lower levels of sTHBS2 at surgery and higher levels of sTHBS2 change were significantly correlated with a better treatment response to NAC. The mean level of sTHBS2 at surgery and absolute sTHBS2 change for patients with RCB-I, RCB-II, and RCB-III was 16.38, 19.53, 22.95 ng/ml and 8.71, 5.15, and 3.87 ng/ml, respectively.

### hTHBS2 and Survival in Non-pCR Patients

**FIGURE 2:**
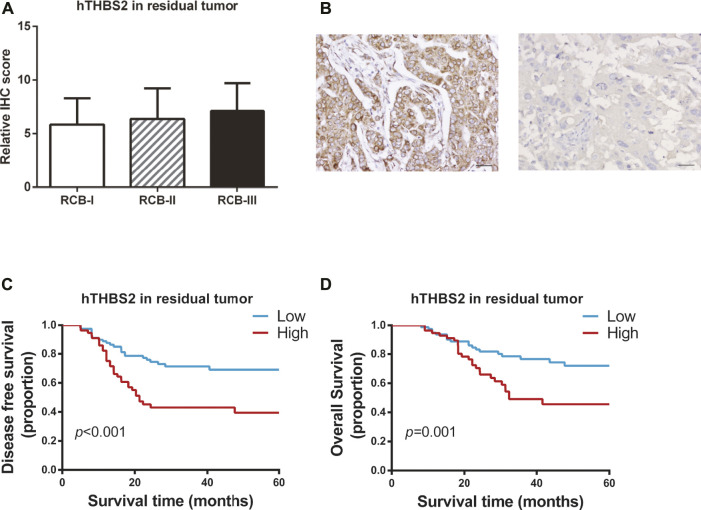
Immunohistochemistry staining of THBS2 in TNBC samples with non-pCR patients after neoadjuvant chemotherapy. **(A)** Immunohistochemistry scores for THBS2 in patients with different RCB grade; **(B)** Representative IHC images of strong and weak THBS2 staining in cytoplasms. (×200); Scale bar: 50 μm. Kaplan-Meier curves of disease-free survival **(C)** and overall survival **(D)** for non-pCR patients according to histological THBS2 expression in non-pCR patients. hTHBS2, histological THBS2.

Of the 185 patients, 48 achieved pCR and 137 were non-pCR responders. Only two patients with pCR were found to have tumor relapse, while 56 (40.9%) cases of non-pCR developed disease recurrence or metastasis. Therefore, survival analyses were conducted to explore the relationship between hTHBS2 expression, clinicopathological factors, and survival of these 137 non-pCR responders. The distributions of hTHBS2 expression in each RCB grade (RCB0, RCB-I, RCB-II, and RCB-III) were displayed in Figure 2A. No statistically significant difference for hTHBS2 was observed within each group. Representative IHC images of strong and weak THBS2 staining in cytoplasms were shown in Figure 2B.

In univariate analysis (Table 2), clinical stage at baseline (*p* = 0.001; *p* = 0.003), residual tumor size (*p* < 0.001; *p* < 0.001), residual lymph nodes (*p* < 0.001; *p* < 0.001), and hTHBS2 expression (*p* < 0.001; *p =* 0.001) were identified to be significant predictors of both disease-free survival and overall survival, respectively. Kaplan-Meier curves of the DFS and OS for the low hTHBS2 and high hTHBS2 groups are presented in Figures 2C,D. Better survival was more frequently observed in patients with a lower expression of hTHBS2 in residual tumor. A multivariate analysis with the cox proportional hazards model was then performed and the relevant results are shown in Table 3. Residual lymph nodes involvement (*p* < 0.001) and hTHBS2 expression in residual tumor (HR = 2.21, 95%CI = 1.24–3.94, *p* = 0.007) were of independent prognostic value for disease-free survival. As for overall survival, residual tumor size (*p* = 0.004), residual lymph nodes involvement (*p* = 0.022), and hTHBS2 expression in residual tumor (HR = 2.07, 95%CI = 1.09–3.92, *p* = 0.026) were identified as independent prognostic factors for patients’ outcome.

**TABLE 2:** Univariate Cox proportional hazard model for disease-free survival (DFS) and overall survival (OS) in non-pCR patients.

Variables	DFS		OS	
	HR (95% CI)	*P* ^a^	HR (95% CI)	*P* ^a^
Age (years)		0.369		0.278
≤50	References		References	
>50	0.78 (0.45–1.34)		0.72 (0.40–1.30)	
Clinical stage at baseline		0.001		0.003
Stage II	References		References	
Stage III	2.51 (1.43–4.40)		2.50 (1.35–4.60)	
Residual tumor size		<0.001		<0.001
ypT0/ypT1	References		References	
ypT2	1.96 (1.10–3.52)		2.08 (1.09–3.97)	
ypT3	4.79 (2.31–9.96)		7.22 (3.33–15.64)	
Residual lymph nodes		<0.001		<0.001
ypN0/ypN1	References		References	
ypN2	4.63 (2.41–8.91)		3.85 (1.93–7.68)	
ypN3	4.62 (2.48–8.62)		3.36 (1.71–6.62)	
Histology		0.992		0.473
Invasive ductal carcinoma	References		References	
Others	1.00 (0.46–2.22)		1.45 (0.52–4.05)	
Ki67 expression at surgery		0.180		0.128
≤50%	References		References	
>50%	1.44 (0.85–2.45)		1.56 (0.88–2.76)	
hTHBS2 expression in residual tumor		<0.001		0.001
Low	References		References	
High	2.73 (1.60–4.71)		2.59 (1.44–4.64)	

Abbreviation: HR, hazard ratio; CI, confidence interval; DFS, disease free survival; OS, overall survival.

a The *p* value was adjusted by the univariate Cox proportional hazard regression model.

**TABLE 3:** Multivariate Cox proportional hazard model for disease-free survival (DFS) and overall survival (OS) in non-pCR patients.

Variables	DFS		OS	
	HR (95% CI)	*P* ^a^	HR (95% CI)	*P* ^a^
Clinical stage at baseline				
Stage II	References	0.055	References	0.056
Stage III	1.81 (0.99–3.33)		1.91 (0.98–3.72)	
Residual tumor size				
ypT0/ypT1	References	0.189	References	0.004
ypT2	1.44 (0.78–2.67)		1.66 (0.84–3.28)	
ypT3	2.10 (0.93–4.75)		4.47 (1.84–10.84)	
Residual lymph nodes				
ypN0/ypN1	References	<0.001	References	0.022
ypN2	4.59 (2.36–8.93)		3.58 (1.76–7.25)	
ypN3	2.58 (1.27–5.23)		1.37 (0.61–3.07)	
hTHBS2 expression in residual tumor				
Low	References	0.007	References	0.026
High	2.21 (1.24–3.94)		2.07 (1.09–3.92)	

Abbreviation: HR, hazard ratio; CI, confidence interval; DFS, disease free survival; OS, overall survival.

a The *p* value was adjusted by the multivariate Cox proportional hazard regression model.

### THBS2 Promoted Proliferation, Migration, and Invasion of TNBC Cells

**FIGURE 3:**
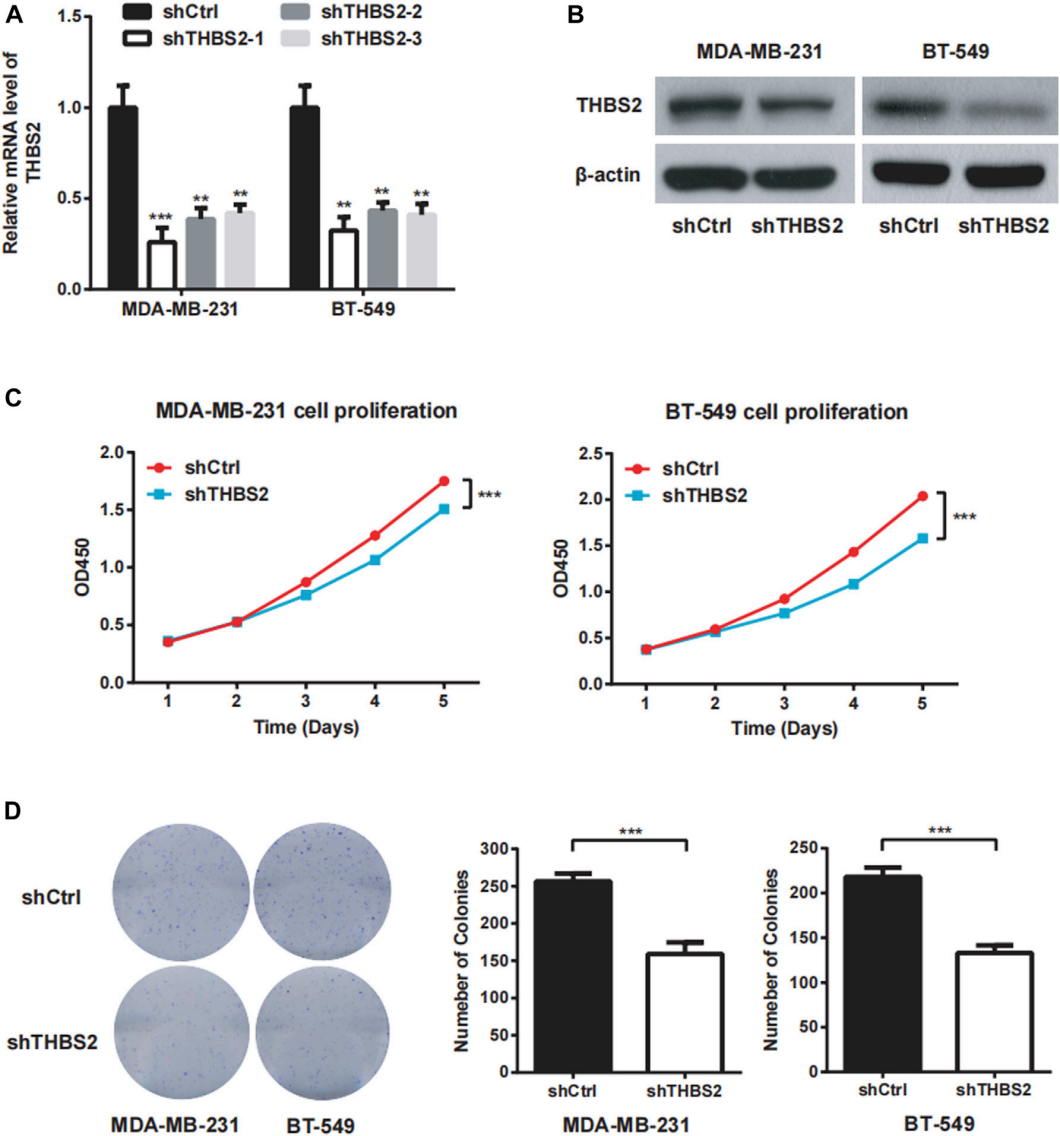
THBS2 promotes cell proliferation in TNBC cells. **(A)** The knockdown efficiency of three shRNA was detected through qRT-PCR in MDA-MB-231 and BT-549 cells. **(B)** The knockdown efficiency of the shRNA for the following experiment was confirmed by western blotting. **(C)** Cell viability was measured by CCK8 assay following THBS2 knockdown in MDA-MB-231 and BT-549 cells. **(D)** Colony formation assay was conducted following THBS2 knockdown in MDA-MB-231 and BT-549 cells. *, *p* < 0.05; **, *p* < 0.01; ***, *p* < 0.001.

**FIGURE 4:**
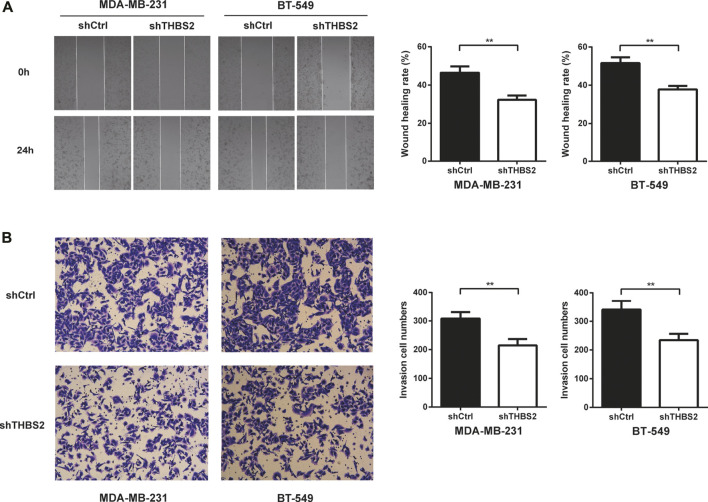
THBS2 promotes TNBC cell migration and invasion in TNBC cells. **(A)** Migration ability was detected by wound healing assay following THBS2 knockdown in MDA-MB-231 and BT-549 cells. **(B)** Invasion ability was detected by transwell invasion assay following THBS2 knockdown in MDA-MB-231 and BT-549 cells. **, *p* < 0.01.

To investigate the function role of THBS2 in TNBC cells, we selected MDA-MB-231 and BT-549 cell lines for further studies. Three specific shRNA (shTHBS2) were designed and synthesized to knock down THBS2 in both cell lines. After stable expression, the shRNA (shTHBS2-1) which exhibited the highest interference efficiency was selected for the following experiments (Figure 3A). Western blot was further conducted to validate the interference efficiency (Figure 3B). The expression of THBS2 in both cells was confirmed to be suppressed compared with the shCtrl group. CCK-8 and colony formation assays revealed that knockdown of THBS2 significantly inhibited the proliferation and colony formation ability for both cells (Figures 3C,D). For wound healing and transwell assays, we also observed a notable decrease migration and invasion potential for THBS2 knockdown cells (Figures 4A,B). Together, these results suggest that THBS2 could act as a tumor activator for the growth and metastasis of TNBC cells.

## Discussion

Triple-negative breast cancer is a heterogeneous disease with high invasiveness. The lack of ER, PR, and HER2 expression renders TNBC unresponsive to endocrine or anti-HER2 target therapy. The basis of TNBC treatment up to now has been a combination of surgery, chemotherapy, and radiotherapy. Anthracycline and taxane-based chemotherapy regimen is the standard of care for prevention of TNBC recurrence and for survival improvement. Currently, the use of neoadjuvant chemotherapy (NAC) has also provided new insight into tumor biology and become a good platform to test drug sensitivity. After completing NAC treatment, TNBC patients have a higher pathological complete response (pCR) rate than those with other subtypes of breast cancer. Patients who achieved pCR could have a lower risk of relapse or death compared to those who have residual disease after NAC [3, 5, 6]. Therefore, prioritizing novel biomarkers for predicting chemotherapy response and promising therapeutic targets might be beneficial in the personalized treatment of TNBC. Relevant studies in the neoadjuvant setting could also help investigators understand the mechanisms of chemotherapy resistance and ultimately improve the outcomes for TNBC patients.

Thrombospondins (THBS) are a family of five secreted matricellular glycoproteins that broadly regulate cell-matrix interaction, angiogenesis, cell proliferation, and apoptosis [26–29]. Among all thrombospondins, THBS2 has been most commonly studied in cancer diagnosis and progression. Circulating levels of THBS2 was confirmed to be a potential diagnostic candidate in pancreatic cancer and lung cancer [15–17, 19], while the histological expression of THBS2 has been identified to be an independent prognostic biomarker for distal cholangiocarcinoma, colorectal cancer, and urothelial carcinoma [18, 22, 30]. Some other studies also noted that THBS2 could act as a useful salivary marker for the detection of oral cavity squamous cell carcinoma [31]. However, few studies have evaluated the predictive and prognostic value of THBS2 on triple-negative breast cancer or in a neoadjuvant setting specifically. Our findings firstly provided evidence that the level of serum THBS2 at surgery and its change during neoadjuvant chemotherapy were able to distinguish TNBC patients who would achieve pathological complete response (OR = 0.88, 95%CI: 0.79–0.98, *p* = 0.020 and OR = 1.12, 95%CI: 1.02–1.23, *p* = 0.015, respectively, as continuous variables). A significant decrease of serum THBS2 after NAC was also observed in patients with lower RCB grade. NAC response is of great importance to TNBC patients in chemotherapy treatment, for an accurate prediction of response might help physicians modify the strategy accordingly. Those patients who are predicted to be non-respondents could be recommended to have an alternative or intensive regimen, thereby improving the outcome of patients with TNBC. Physical and imaging examinations are two of the most common response evaluation methods for patients in neoadjuvant chemotherapy treatment. Numerous studies have implied that magnetic resonance imaging (MRI) could act as an effective tool for predicting pCR, especially in TNBC patients [32–34]. However, the accuracy might be lower when pCR is more rigorously defined [35]. The results of our study have confirmed that the secretion of THBS2 during chemotherapy was correlated with chemotherapy resistance. The predictive role of THBS2 might be an easier and more valid tool to identify patients who would benefit more in NAC. Some other biomarkers, such as vascular endothelial growth factor (VEGF), soluble programmed death 1 (sPD-1), and soluble programmed death ligand-1 (sPD-L1), have also been indicated in similar studies [36, 37]. A combination of these serum biomarkers and radiomic metrics might provide more precise information for TNBC patients with NAC.

For triple-negative breast cancer, residual tumors are often heterogeneous and believed to represent chemotherapy resistant micro-metastatic disease which could ultimately evolve into clinical metastatic breast cancer [38]. Identification of patients who are at high risk of relapse after neoadjuvant chemotherapy could guide more appropriate post-neoadjuvant treatment strategies. In this study, we also provided evidence that the histological THBS2 (hTHBS2) expression in residual tumor was independently associated with disease-free survival and overall survival for TNBC non-pCR responders (HR = 2.21, 95%CI = 1.24–3.94, *p* = 0.007 and HR = 2.07, 95%CI = 1.09–3.92, *p* = 0.026, respectively). In addition, the expression of hTHBS2 was found to have no correlation with different TNBC subtypes (LAR, IM, BLIS, MES, and US) or RCB grade. Higher expression of hTHBS2 might reflect a group of TNBC tumors with more aggressive behavior and poor outcome. For now, the evaluation of residual disease after NAC is mainly based on the tumor regression system (quantitative amount of tumor), such as the RCB categories, Miller-Payne (MP) grade, or Neo-Bioscore [39–41], while a qualitative method for distinguishing different TNBC subsets and exploration of proper prognostic biomarkers in the post-NAC settings could also be a novel direction in the future.

In addition to its predictive and prognostic value in TNBC patients with neoadjuvant chemotherapy, we also investigated the function role of THBS2 in MDA-MB-231 and BT-549 cells. CCK-8 and colony formation assays demonstrated that knockdown of THBS2 significantly inhibited the proliferation and colony formation ability for both cells. In addition, wound healing and transwell assays showed notable decrease migration and invasion potential for THBS2 knockdown cells. Numerous studies have strongly suggested that THBS2 is an oncogene and involved in the malignant phenotype of different tumors, such as gastric cancer [42], lung cancer [12], and osteosarcoma [43]. Our findings have also confirmed its significant role in promoting TNBC proliferation, migration, and invasion. However, there are several limitations which should also be mentioned in our study. Firstly, the sample size of this study was still limited to establish an external validation cohort. Future multi-institutional and larger studies are still warranted to validate our results. Secondly, we only collected serum sample and evaluated the sTHBS2 level at two time points (at baseline and surgery). Relative serum samples of each two cycles of neoadjuvant chemotherapy were not obtained and analyzed. An earlier detection of sTHBS2 change and more accurate mid-term evaluation could help modify the treatment regimen promptly and avoid unnecessary treatment-related toxicities. Lastly, the study of the regulatory mechanism of THBS2 in TNBC is inadequate and needs further in-depth research.

## Conclusion

In summary, we revealed a novel strategy for response evaluation and prediction for TNBC patients with neoadjuvant chemotherapy. THBS2 might serve as a promising functional predictive and prognostic biomarker for patients with triple-negative breast cancer.

## Data Availability

The raw data supporting the conclusion of this article will be made available by the authors, without undue reservation.
