# Supplementary material for: Thrombospondin 2 is a Functional Predictive and Prognostic Biomarker for Triple-Negative Breast Cancer Patients With Neoadjuvant Chemotherapy
Source: Pathol Oncol Res. 2022 Aug 30;28:1610559. doi: 10.3389/pore.2022.1610559 (PMC9673122; doi:10.3389/pore.2022.1610559)
Supplement: Supplementary file 1 [file Table1.docx]

**Supplementary Table 1** Sequences of shRNAs and primers used in this study.

| **Primer** | **Sequence** |
| --- | --- |
| shTHBS2-1 | 5'-CTGCGACCTCATAGACAGCTT-3' |
| shTHBS2-2 | 5'-CCGCTTCGTGCGCTTTGACTA-3' |
| shTHBS2-3 | 5'-TTGCTTCAGAACGTCCACCTA-3' |
| shCtrl | 5'-TTCTCCGAACGTGTCACGT-3' |
| THBS2-F | 5’-GGTGGCTGGCGAGACCTACA-3’ |
| THBS2-R | 5’-TGGCAACCCTTCTTGCTTAG-3’ |
| β-actin-F | 5’-GCGTGACATTAAGGAGAAGC-3’ |
| β-actin-R | 5’-CCACGTCACACTTCATGATGG-3’ |
